# Myo-Inositol Transporter SLC5A3 Associates with Degenerative Changes and Inflammation in Sporadic Inclusion Body Myositis

**DOI:** 10.3390/biom10040521

**Published:** 2020-03-30

**Authors:** Boel De Paepe, Caroline Merckx, Jana Jarošová, Miryam Cannizzaro, Jan L. De Bleecker

**Affiliations:** Department of Neurology; Laboratory for Neuropathology, Ghent University Hospital, Corneel Heymanslaan 10, 9000 Ghent, Belgium; caroline.merckx@ugent.be (C.M.); jaroja1@seznam.cz (J.J.); miryamcan@gmail.com (M.C.); jan.debleecker@ugent.be (J.L.D.B.)

**Keywords:** sporadic inclusion body myositis, myo-inositol, protein aggregation, inflammation

## Abstract

Myo-inositol exerts many cellular functions, which include osmo-protection, membrane functioning, and secondary messaging. Its Na^+^/myo-inositol co-transporter SLC5A3 is expressed in muscle tissue and further accumulates in myositis. In this study we focused on the peculiar subgroup of sporadic inclusion body myositis (IBM), in which auto-inflammatory responses and degenerative changes co-exist. A cohort of nine patients was selected with clinically confirmed IBM, in which SLC5A3 protein was immune-localized to the different tissue constituents using immunofluorescence, and expression levels were evaluated using Western blotting. In normal muscle tissue, SLC5A3 expression was restricted to blood vessels and occasional low levels on muscle fiber membranes. In IBM tissues, SLC5A3 staining was markedly increased, with discontinuous staining of the muscle fiber membranes, and accumulation of SLC5A3 near inclusions and on the rims of vacuoles. A subset of muscle-infiltrating auto-aggressive immune cells was SLC5A3 positive, of which most were T-cells and M1 lineage macrophages. We conclude that SLC5A3 is overexpressed in IBM muscle, where it associates with protein aggregation and inflammatory infiltration. Based on our results, functional studies could be initiated to explore the possibilities of therapeutic osmolyte pathway intervention for preventing protein aggregation in muscle cells.

## 1. Introduction

The carbocyclic sugar myo-inositol (MI) ensures the smooth running of many cellular functions. Firstly, MI constitutes a component of membrane phospholipids required for correct membrane functioning. Secondly, MI is one of the most abundant stabilizing osmolytes in mammalian cells, protecting from osmotic cell stress and from inappropriate cell shrinkage or volume increase. When cells experience extracellular hypertonicity, the expression of MI carriers is upregulated via activation of the transcription factor nuclear factor of activated T-cells 5 (NFAT5), ensuring further accumulation of MI to uphold the cell’s osmotic balance. Two MI transporters belong to solute carrier family 5, a group of membrane transport proteins of which the importance is stressed by the many genetic and acquired human diseases they associate with [1]. Na^+^/myo-inositol co-transporters 1 (SLC5A3) and 2 (SLC5A11) co-import 2 Na^+^ with MI from the extracellular fluid and are widely expressed throughout the body. SCL5A3 is of critical importance in the developing nervous system, the SLC5A11 transporter has been proposed as a modifier for immune-related genes [2]. A third MI carrier, the H^+^/myo-inositol co-transporter SLC2A13, is strongly expressed in the central nervous system [3]. Thirdly, MI is a precursor of inositol triphosphate, an important second messenger that regulates a plethora of intracellular processes. Phosphatidylinositol 3-kinase signaling ensures, among other processes, cell growth, proliferation and survival, and immune homeostasis [4]. In the human body, highest levels of MI can be detected in brain, kidney, spleen, stomach, liver, heart, and skeletal muscle. The main source of MI is through import from the extracellular medium by its designated carriers. MI can also be synthetized inside the cell starting from glucose 6-phosphate, a process most active in the kidney [5].

Inflammatory myopathies comprise diseases of idiopathic origin that are characterized by chronic muscle inflammation and weakness. They are considered autoimmune disorders in which the body’s immune response targets muscle fibers, blood vessels, connective tissue, organs, and/or the joints. Within the group of idiopathic inflammatory myopathies, the sporadic form of inclusion body myositis (IBM) represents a disorder with peculiar features [6]. It is a slowly progressive condition with onset in middle or late age, with a complex and multifaceted pathogenesis [7]. IBM is associated with changes of metabolism and mitochondrial dysfunction characterized by abnormal mitophagy, mitochondrial DNA alterations, and oxidative phosphorylation defects [8]. Degenerative damage accumulates in IBM muscle tissues at a higher pace than observed in normal muscle aging. Subsets of muscle fibers accumulate aberrant structures, among which are characteristically rimmed vacuoles and inclusions composed of granular basophilic material. Misfolded and aggregated proteins form complexes and β-sheet-rich fibrils, resembling the β-amyloid plaques in Alzheimer’s disease, and the α-synuclein bodies in Parkinson’s disease. Alongside the neurodegenerative features, the immune component of IBM is conspicuous. Muscle fibers are actively invaded by auto-aggressive cytotoxic cells, and circulating autoantibodies are present in subsets of patients [9].

We reported earlier that the osmolyte system becomes activated in myositis tissues [10] and in vitro in muscle cells exposed to pro-inflammatory cytokines [11]. In the present study we elaborate on the involvement of the MI transporter SLC5A3 in the degenerative and inflammatory changes associated with IBM.

## 2. Materials and Methods

### 2.1. Patients

For participation in this study, nine patients with idiopathic inflammatory myopathy classified as IBM were selected from the files of the Ghent University Hospital (Table 1). Commercial multiplex line blot assays checked the presence of common myositis-specific and myositis-associated autoantibodies in samples from patients 1 to 3 and 7 to 9, which were all negative. Additionally, enzyme-linked immunosorbent assay detected anti-cN1A antibodies in patients 1 and 9 using a protocol described previously [12]. Signals were quantified by determining optical densities at 450 nm, with sera assessed as reactive if above the established cutoff value for at least one of the peptide antigens. Ten control subjects were selected that were age matched. These controls were patients that underwent a diagnostic muscle biopsy, but in whom no evidence was found of an underlying muscle disease, nor any clinical manifestations that were suggestive of an inflammatory myopathy. Sampling adhered to ethical standards and privacy regulations following procedures approved by the Ghent University Hospital Ethics Committee (B670201836756, B670201938779). All subjects signed informed consent for the scientific use of their anonymized materials.

### 2.2. Antibodies for Immunodetection

All antibodies used were commercially available and are listed in Table 2.

### 2.3. Immunofluorescence

SLC5A3 was visualized in 8 µm frozen muscle sections, that were blocked with phosphate buffered saline containing 5% donkey serum, 10% heat-inactivated human serum, and 2% bovine serum albumin. Double staining was done with a mouse monoclonal antibody directed against inclusions and protein aggregates, and specific monoclonal antibodies for immune cell types. Immunofluorescent staining was done for all 9 patients, stained with both anti-SLC5A3 antibodies, and in combinations with all five marker antibodies. Fluorescent secondary antibodies were donkey anti-rabbit labeled with CY3 (Jackson ImmunoResearch Laboratories, West Grove, PA, USA) and donkey anti-mouse labeled with AlexaFluor488 (Invitrogen, Carlsbad, CA, USA). Slides were mounted with Fluoromount (Southern Biotech, Birmingham, AL, USA) and analyzed under a fluorescence microscope (Zeiss, Goettingen, Germany). Negative controls consisted of the omission of primary antibody or their substitution by non-immune IgGs. Specificity of SLC5A3 staining was verified by staining human kidney sections as a positive control tissue. Omitting the primary antibody resulted in no background staining.

### 2.4. Western Blotting

From three patients (IBM1-3), sufficient muscle material was available to prepare protein samples, which was carried out using a commercial extraction kit (Thermo Fisher Scientific, Waltham, MA, USA). A positive control sample was prepared from a human kidney biopsy. Proteins were separated on a 10% bis-tris gel and blotted onto a nitrocellulose membrane (Thermo Fisher Scientific). The membrane was incubated simultaneously with primary antibodies directed against SLC5A3 and glyceraldehyde 3-phosphate dehydrogenase (GAPDH), in tris-buffered saline with 2% milk added. Chemiluminescence was generated after incubating for 1h with 1µg/mL peroxidase-labeled rabbit anti-mouse by adding the Clarity Max substrate (Biorad, Hercules, CA, USA), visualized with the Chemidoc Imaging System, and analyzed with Image Lab 6.0 software (Biorad). Quantification was done using GeneTools software (Syngene, Cambridge, UK), automatically detecting and measuring raw volume of protein bands. SLC5A3 levels were calculated as the raw volume relative to the GAPDH content of the sample.

## 3. Results

**Figure 1:**
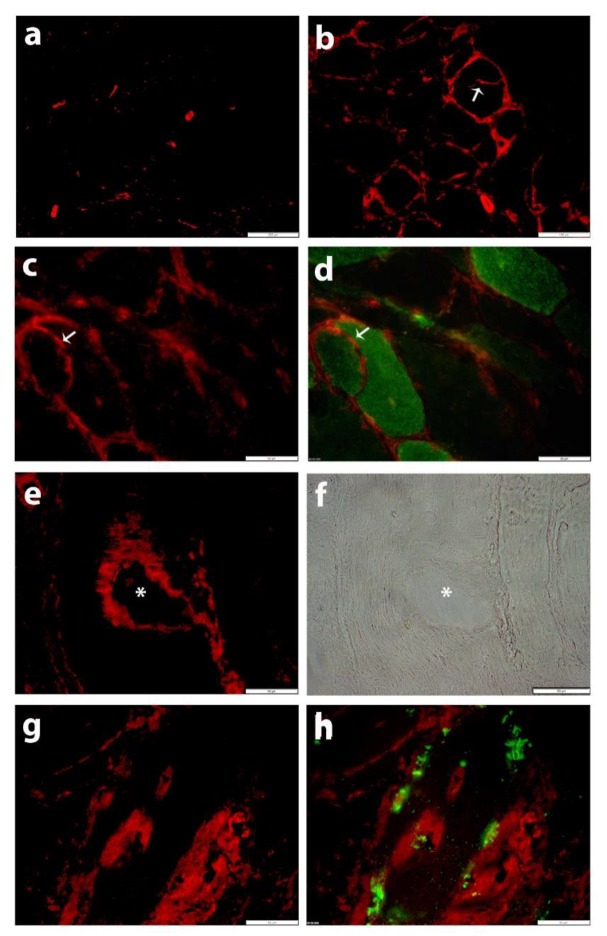
Immunofluorescent staining for SLC5A3 (CY3, red) with antibody Ab1. (**a**) In normal skeletal muscle tissue, staining is restricted to blood vessels and occasional partial staining of muscle fiber membranes. (**b**–**h**) SLC5A3 staining (CY3, red) is increased in the muscle tissue from patient IBM7: (**b**) SLC5A3 staining is observed on blood vessels, endomysial inflammatory cells and the muscle fiber membrane, including the membrane of a splitting fiber (arrow), which is a pathological feature associated with muscle tissue damage and regeneration. (**c**,**d**) Discontinuous membranous SLC5A3 staining is present on a small fiber that is CD56+ (AlexaFluor488, green, arrow). (**e**) SLC5A3 staining is accentuated on the rims of a vacuole, as confirmed by the phase contrast image (**f**, asterisk). (**g**,**h**) SLC5A3 staining associates with the presence of inclusions, visualized as sequestosome 1/p62-positive (AlexaFluor488, green). Scale bars 200 µm (**a**), 100 µm (**b**), 50 µm (**c**–**h**).

The two rabbit polyclonal anti-SLC5A3 antibodies showed similar immunofluorescent staining patterns in human skeletal muscle sections. In normal skeletal muscle tissues, SLC5A3 staining was restricted to blood vessels and occasional partial staining of the muscle fiber membrane (Figure 1a). In IBM tissues, SLC5A3 expression became markedly upregulated. Discontinuous SLC5A3 expression was present on muscle fiber membranes (Figure 1b,c). Discontinuous membranous SLC5A3 staining was often observed on CD56+ muscle fibers, representing regenerating or denervated muscle fibers (Figure 1c,d). In addition, SLC5A3 staining was detected inside IBM muscle fibers, where the transporter localized to vacuoles (Figure 1e,f) and associated with the presence of sequestosome 1/p62+ inclusions (Figure 1g,h). In comparison, infrequent partial staining of the muscle fiber membrane was observed with an antibody directed against SLC5A11, which was not different in normal controls and in IBM tissues (Supplementary Figure S1).

**Figure 2:**
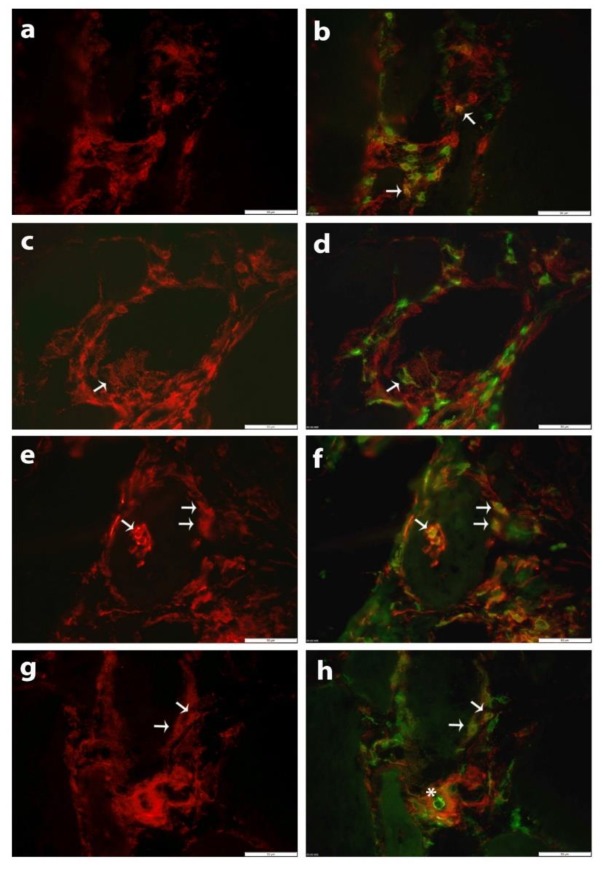
Immunofluorescent staining of SLC5A3 (CY3, red) with antibody Ab2 in skeletal muscle tissue from patient IBM5 (**a**–**h**). (**b**) Double stain with anti-CD3 (AlexaFluor488, green) shows partial co-localization (arrows). (**d**) Double stain with anti-CD206 (AlexaFluor488, green) marks a cell that is weakly SLC5A3 positive (arrow). (**f**) Double staining with anti-CD68 (AlexaFluor488, green) shows part of these cells are strongly positive, mostly cells invading and surrounding a non-necrotic fiber (arrows). (**h**) A CD68+ cell (AlexaFluor488, green) adherent to the luminal side of a blood vessel is negative (asterisk), while subsets of endomysial CD68+ cells express SLC5A3 (arrows). Scale bars 50 µm in all panels.

Specific subsets of muscle-infiltrating inflammatory cells expressed SLC5A3. Many T-cells, marked as CD3+, were SLC5A3 positive (Figure 2a,b). CD206 + M2 lineage macrophages were mostly SLC5A3 negative, and those stained showed low levels of SLC5A3 (Figure 2c,d). More abundant SLC5A3 positivity was observed in CD68+ cells of which a large proportion are known to represent macrophages of the M1 phenotypic lineage. Endomysial CD68+ cells, and cells invading muscle fibers were often SLC5A3 positive (Figure 2e–f), while cells that adhered to the blood vessel cell wall at the luminal side were SLC5A3 negative (Figure 2g–h). This staining pattern closely resembled that of polymyositis tissues, while markedly lower numbers of SCL5A3 positive immune cells were present in dermatomyositis and immune-mediated necrotizing myopathy [10].

**Figure 3:**
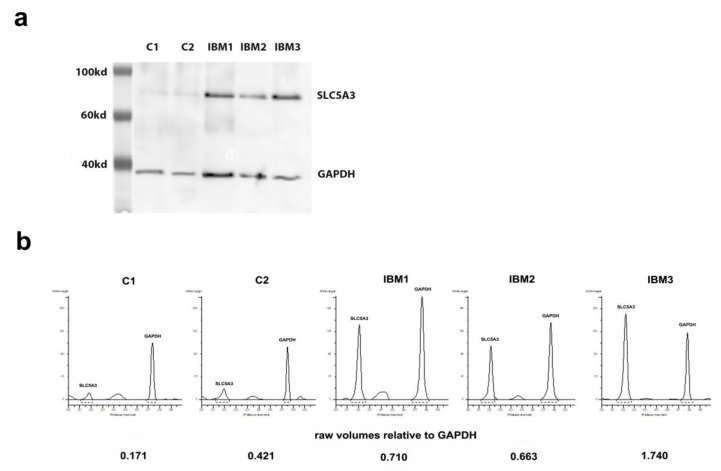
(**a**) Western blot visualizations of SLC5A3 with the Ab3 antibody, detected simultaneously with anti-glyceraldehyde-3-phosphate dehydrogenase (GAPDH), the latter as a control for differences in protein densities of samples. Note a very faint SLC5A3 protein band in controls (C) and prominent bands in muscle samples from patients with sporadic inclusion body myositis (IBM). (**b**) Raw volumes of protein bands as quantified using GeneTools software, with SLC5A3 density calculated relative to the GAPDH level of the sample.

In the human kidney sample, abundant SLC5A3 expression could be shown as a strong single protein band of approximately 80kd using Western blotting (data not shown). SLC5A3 protein was barely detectable in control muscle, while a strong protein band was present in the three IBM muscle samples that were tested (Figure 3).

## 4. Discussion

Our study shows that SLC5A3 is upregulated in IBM muscle and that the osmolyte carrier localizes to the muscle fibers, blood vessels, and inflammatory cells. Overexpressed SLC5A3 has also been observed in other inflammatory myopathies and in muscular dystrophies [11]. Yet, the co-localization with the muscle fiber inclusions is peculiar for IBM, a muscle disease in which degenerative and inflammatory characteristics are equally prominent.

When neurodegenerative processes lead to the disruption of protein folding homeostasis, osmolytes appear to exhibit compensatory effects. The upregulation of the MI transporter SLC5A3 in IBM that we report here presumably represents an appropriate response to slow down the accumulation of aberrant proteins. These results mimic findings of elevated MI repeatedly observed in Alzheimer’s disease, where the osmolyte levels associate with β-amyloid plaque deposition in asymptomatic [13] and symptomatic [14] patients. A true mechanistic relationship between the two phenomena is suggested by the observation that plaque-removing immunization intervention attenuates MI levels in a transgenic murine model for Alzheimer’s [15]. Attenuated toxicity of the deposits has been described as originating from β-amyloid stabilization by complex formation with MI [16], preventing lateral stacking of protofibrillar β-sheet oligomers [17]. The osmolyte pathway therefore may represent an amenable therapeutic strategy for treating degenerative disorders. There are, however, fundamental differences between the insoluble β-amyloid plaques in between nerve cells associated with Alzheimer’s disease, and the intracellular β-amyloid-containing inclusions in IBM muscle fibers. The feature of intracellular neurofibrillar tangles of tau protein that have their equivalent in the vacuolated muscle fibers containing paired-helical filaments in IBM [18] may offer a more direct comparison between the degenerative processes at play in these diseases.

There is also another side to the story, as in IBM degenerative changes are accompanied by inflammatory changes. In this light, the known involvement of osmolyte pathways in chronic inflammation is of interest, and we report SLC5A3 on subsets of muscle tissue-infiltrating immune cells in IBM. The transporter was differentially expressed on macrophages in relation to their different functionalities: less abundant on restorative M2 lineage macrophages, more abundant on M1 cytotoxic lineage macrophages. This nicely fits with the established role for osmolytes in cytotoxic processes, regulated via the upstream transcription factor NFAT5. In T-cells [19] and cytotoxic macrophages [20,21], NFAT5 activation causes MI to accumulate via MAPK-regulated upregulation of SLC5A3. NFAT5-regulated expression profiles favor M1-related immune responses. In murine models of cancer, MI supplements result in increased numbers of anti-tumorigenic M1 macrophages, which benefits survival [22,23]. In view of the auto-aggressive immune cells present in IBM muscle, in this case such pro-inflammatory processes would, on the contrary, be sustaining the chronic inflammation. In addition, osmolyte pathways could be relevant to the recruitment of circulating immune cells to the tissues, through interactions between NFAT5 and the master regulator of inflammation nuclear factor κB (NFκB) [24]. We found intramuscular blood vessels were SLC5A3 positive, which is in line with MI regulating endothelial cell function [25]. In response to pro-inflammatory cytokines, endothelial cells are known to activate NFAT5, leading to the release of the chemoattractant CCL2 [26], an important recruiter of monocytes in IBM [27]. Endothelial CCL2 marks sites of invasion for circulating immune cells, and we speculate that recruited monocytes might be conditioned to acquire SLC5A3 upon invasion of the skeletal muscle tissue. We need to acknowledge also that the inflammatory changes observed in IBM are not separate from the degenerative changes. It has been established that pro-inflammatory cytokine treatment is a potent inducer of the accumulation of aberrant proteins in muscle cells in vitro [28].

For treating neurodegenerative disorders, administering compounds that prevent protein aggregation has been proposed as a viable therapeutic strategy [29]. Osmolytes have the capacity to stabilize proteins, by shifting the equilibrium toward the folded native conformation, away from the unfolded state [30]. The inositol stereoisomer ELND005 decreases in a dose-dependent way amyloid pathology and plaque accumulation in TgCRND8 amyloid precursor protein transgenic mice [31]. Trials evaluating the benefits of MI analogues are ongoing in Alzheimer’s disease [32]. Based upon our presented results, therapies targeting osmolyte pathways might also be worth exploring for IBM. This is of importance as most IBM patients are refractory toward immunosuppressive therapy, and there is at present no effective treatment available for the condition.

## 5. Conclusions

This study identified SLC5A3 as an associated factor and a pathological hallmark in IBM, and proposes osmolyte pathway intervention as an alternative approach to be further explored for future therapeutic management for this currently incurable disorder.

## Figures and Tables

**Table 1:** Sporadic inclusion body myositis (IBM) patient characteristics.

Patient	Gender/Age	Clinical Features	Muscle Pathology	Autoantibodies
IBM1	M/63	Forearm, finger, and quadriceps weakness.	Several non-necrotic invaded fibers and fibers with rimmed vacuoles and inclusions.	cNIA+
IBM2	M/71	Forearm and finger flexor weakness.	Several muscle fibers with rimmed vacuoles and inclusions, rare inflammation.	cNIA-
IBM3	M/72	Forearm, finger, and quadriceps weakness.	Some fibers with rimmed vacuoles and with inclusions.	cNIA-
IBM4	M/70	Quadriceps muscle weakness.	Severe inflammation and fibrosis, many fibers with inclusions.	ND
IBM5	M/65	Forearm flexor and quadriceps weakness.	Severe inflammation, many fibers with inclusions.	ND
IBM6	F/59	Proximal leg and distal finger flexor muscle weakness.	Many non-necrotic invaded fibers, some fibers with inclusions, mild fibrosis.	ND
IBM7	M/83	Forearm flexor and quadriceps weakness.	Several non-necrotic invaded fibers and fibers with rimmed vacuoles and inclusions.	cNIA-
IBM8	F/59	Dysphagia, forearm finger flexor, and quadriceps weakness.	Rare non-necrotic invaded fibers, few inclusions, mild fibrosis.	cNIA-
IBM9	M/72	Quadriceps muscle weakness.	Many fibers with rimmed vacuoles and inclusions, scarce inflammation.	cNIA+

cytosolic 5′-nucleotidase 1A autoantibody seropositive (cNIA+); seronegative (cNIA-); not determined (ND).

**Table 2:** Antibodies used for immunofluorescence (IF) and Western blotting (WB).

Antibody	Antigen	Species and Specifications	Concentration	Reference	Source
Ab1	SLC5A3	rabbit anti-AA221-270	IF 2.5 µg/mL	B4872/27459	LS-Bio
Ab2	SLC5A3	rabbit anti-central region	IF 10 µg/mL	C358323/125465	LS-Bio
Ab3	SLC5A3	mouse anti-AA533-642 IgG_1_	WB 0.8 µg/mL	SAB1402920	Sigma
Ab4	GAPDH	mouse IgG_1_	WB 0.2 µg/mL	B9310/127452	LS-Bio
Ab5	SLC5A11	rabbit polyclonal IgG	IF 4 µg/mL	HPA035331	AtlasAntibodies
Ab6	CD3	mouse IgG_1_	IF 10 µg/mL	M7254	Dako
Ab7	CD56	mouse IgG_1_	IF 5 µg/mL	MAB2120Z	Millipore
Ab8	CD68	mouse IgG_1_	IF 10 µg/mL	Ab955	Abcam
Ab9	CD206	mouse IgG_1_	IF 0.4 µg/mL	MCA2155	Biorad
Ab10	SQSTM1	mouse anti-AA257-437 IgG_1_	IF 1.3 µg/mL	610833	BDTransduction

Na^+^/myo-inositol cotransporter 1/solute carrier A3 (SLC5A3); glyceraldehyde-3-phosphate dehydrogenase (GAPDH); sequestosome 1/p62 (SQSTM1).

## Supplementary Materials

The following are available online at https://www.mdpi.com/2218-273X/10/4/521/s1, Figure S1: Immunofluorescence for SLC5A11.
