# Supplementary material for: Myo-Inositol Transporter SLC5A3 Associates with Degenerative Changes and Inflammation in Sporadic Inclusion Body Myositis
Source: Biomolecules. 2020 Mar 30;10(4):521. doi: 10.3390/biom10040521 (PMC7226596; doi:10.3390/biom10040521)
Supplement: Supplementary file 1 [file biomolecules-10-00521-s001.pdf]

Supplementary Figure S1.

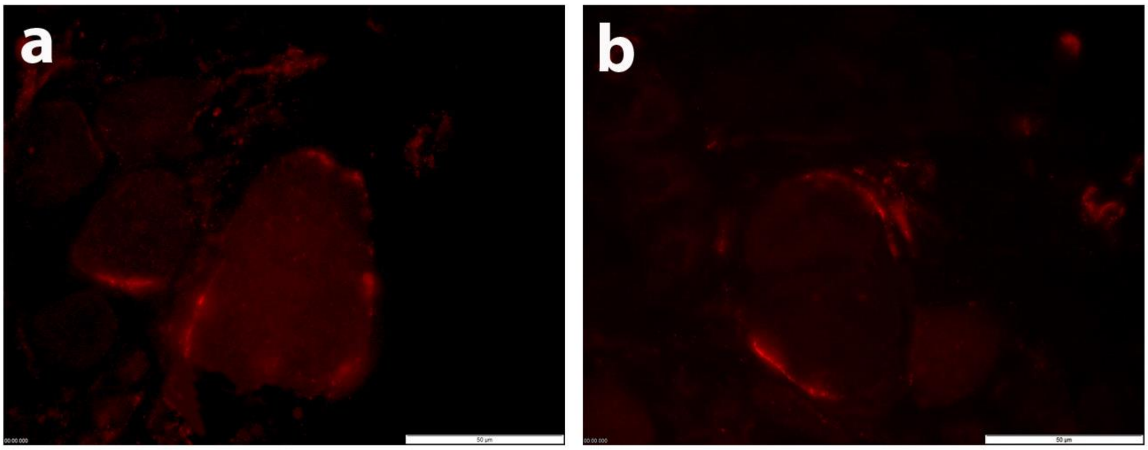

Immunofluorescence for SLC5A11 (CY3, red) presents as similar discontinuous membrane staining on muscle fibers in skeletal muscle tissue from a normal control (**a**) and from sporadic inclusion body myositis patient IBM9 (**b**). Scale bars 50µM.
